# Supplementary material for: Identification of photoperiod-induced specific miRNAs in the adrenal glands of Sunite sheep (Ovis aries)
Source: Front Vet Sci. 2022 Jul 22;9:888207. doi: 10.3389/fvets.2022.888207 (PMC9354845; doi:10.3389/fvets.2022.888207)
Supplement: Supplementary file 1 [file Data_Sheet_1.ZIP › Supplementary file/miRDeep2 file.zip!/miRDeep2 file/Know_Structure/SP42A1/oar-mir-758.pdf]

miRBase precursor	: oar-mir-758
Total read count	: 258
oar-miR-758-5p read count	: 1
oar-miR-758-3p read count	: 257
remaining reads	: 0

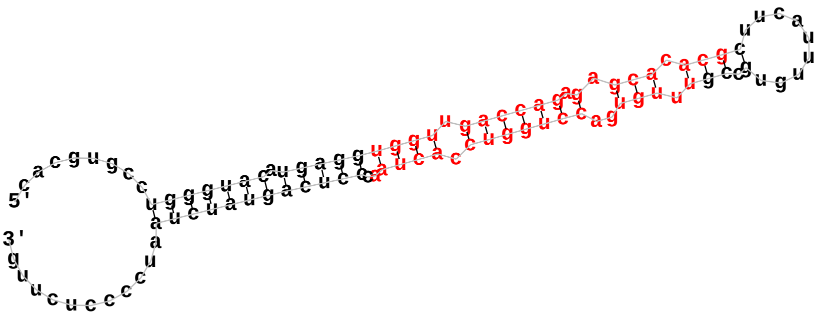

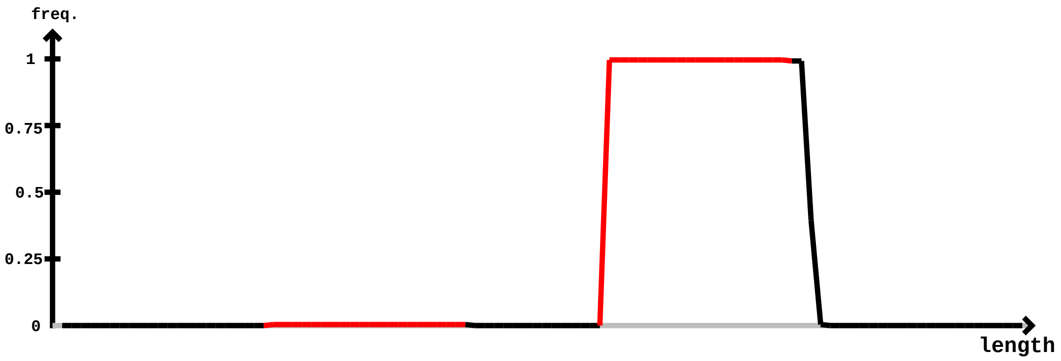


```
                                                        oar-miR-758-3p
                        oar-miR-758-5p
5'- cacgugccuggguacaugaggugguugaccagagagcacacgcuucauuugugccguuugugaccugguccacuaacccucaguaucuaauccccucuug   -3'   exp
    ........(((((((.(((((((((.((((((.(.(((.((((.........).))).))).))))))).)))).))..))))))))))...........   reads   mm   sample
    .....................ugguugaccagagagcacacg..........................................................   1       0    seq
    ........................................................uuugugaccugguccacua.........................   1       0    seq
    ........................................................uuugugaccugguccacuaac.......................   154     0    seq
    ........................................................uuugugaccugguccacuaacc......................   101     0    seq
    ........................................................uuugugaccugguccacuaaccc.....................   1       0    seq
```
